# Supplementary material for: Bidirectional quantitative scattering microscopy
Source: Nat Commun. 2025 Nov 14;16:9712. doi: 10.1038/s41467-025-65570-w (PMC12618536; doi:10.1038/s41467-025-65570-w)
Supplement: Supplementary file 1 — Supplementary Information [file 41467_2025_65570_MOESM1_ESM.pdf]

Supplementary information for
Bidirectional quantitative scattering microscopy

Kohki Horie[1+], Keiichiro Toda[2+], Takuma Nakamura[2] and Takuro Ideguchi[1,2,*]

[1] Department of Physics, The University of Tokyo, Tokyo, Japan

[2] Institute for Photon Science and Technology, The University of Tokyo, Tokyo, Japan

[+]These authors contributed equally to this work

[*] Corresponding author: ideguchi@ipst.s.u-tokyo.ac.jp

Supplementary Note 1: Detailed schematic of bidirectional quantitative scattering microscopy (BiQSM).
Figures S1 and S2 represent a detailed schematic of a visible light source and microscopy setup in BiQSM system, respectively.

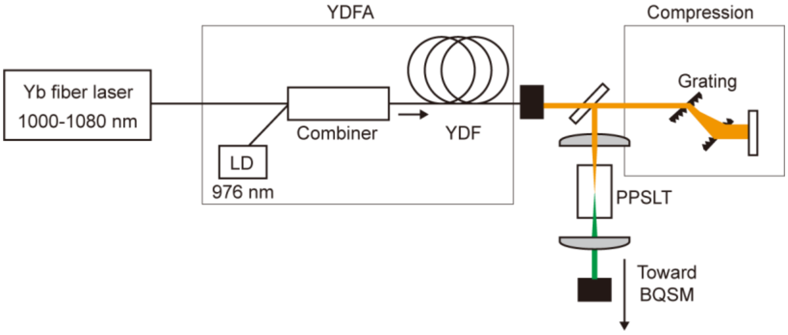

Fig. S1 Schematic of a visible light source in BiQSM system. Yb: ytterbium. YDF: ytterbium-doped fiber. YDFA: ytterbium-doped fiber amplifier. LD: laser diode. PPSLT: periodically poled stoichiometric lithium tantalate.

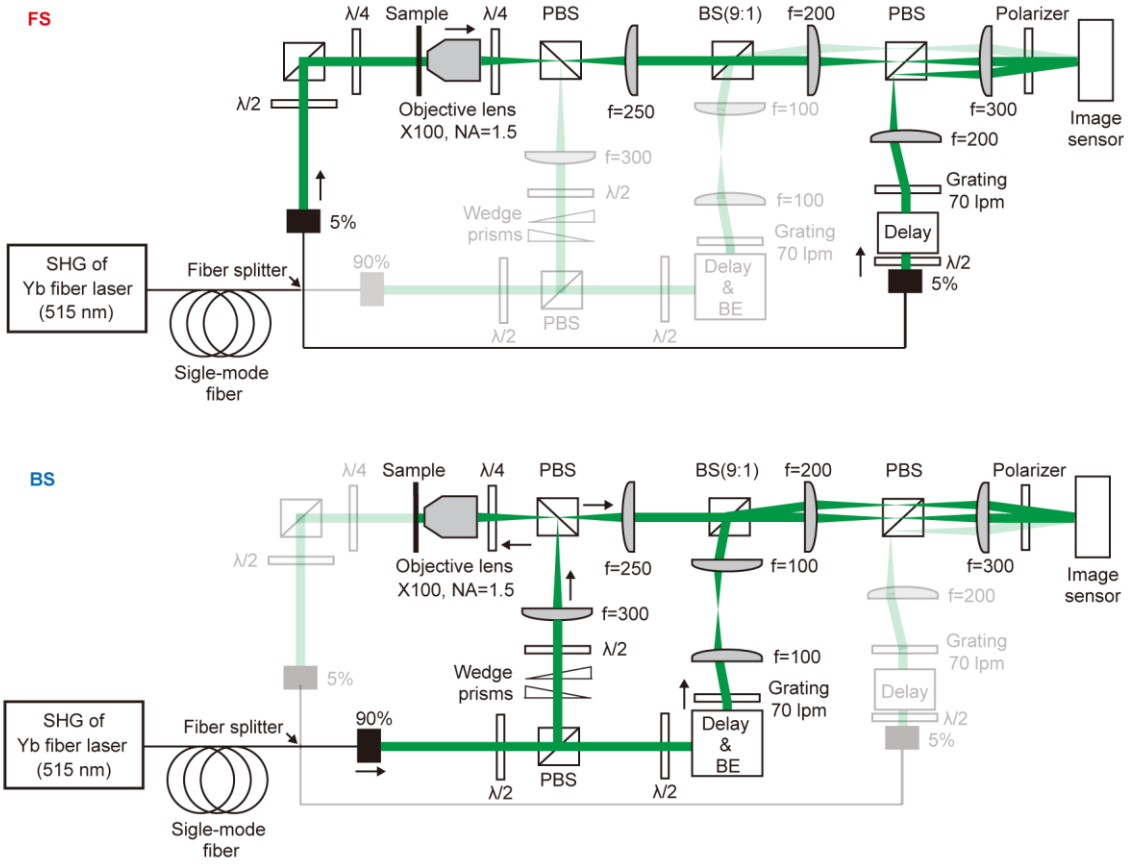

Fig. S2 Schematic of BiQSM system. FS (top) and BS (bottom) imaging system. SHG: second harmonic generation. λ/4 and λ/2: quarter- and half-wave plate. PBS: polarizing beamsplitter. BE: beam expander.

Supplementary Note 2: Computational workflow for generating SA images.

Figure S3 illustrates the comprehensive workflow for generating SA images. Detailed explanations are provided in the “SA calculation procedure” in Methods.

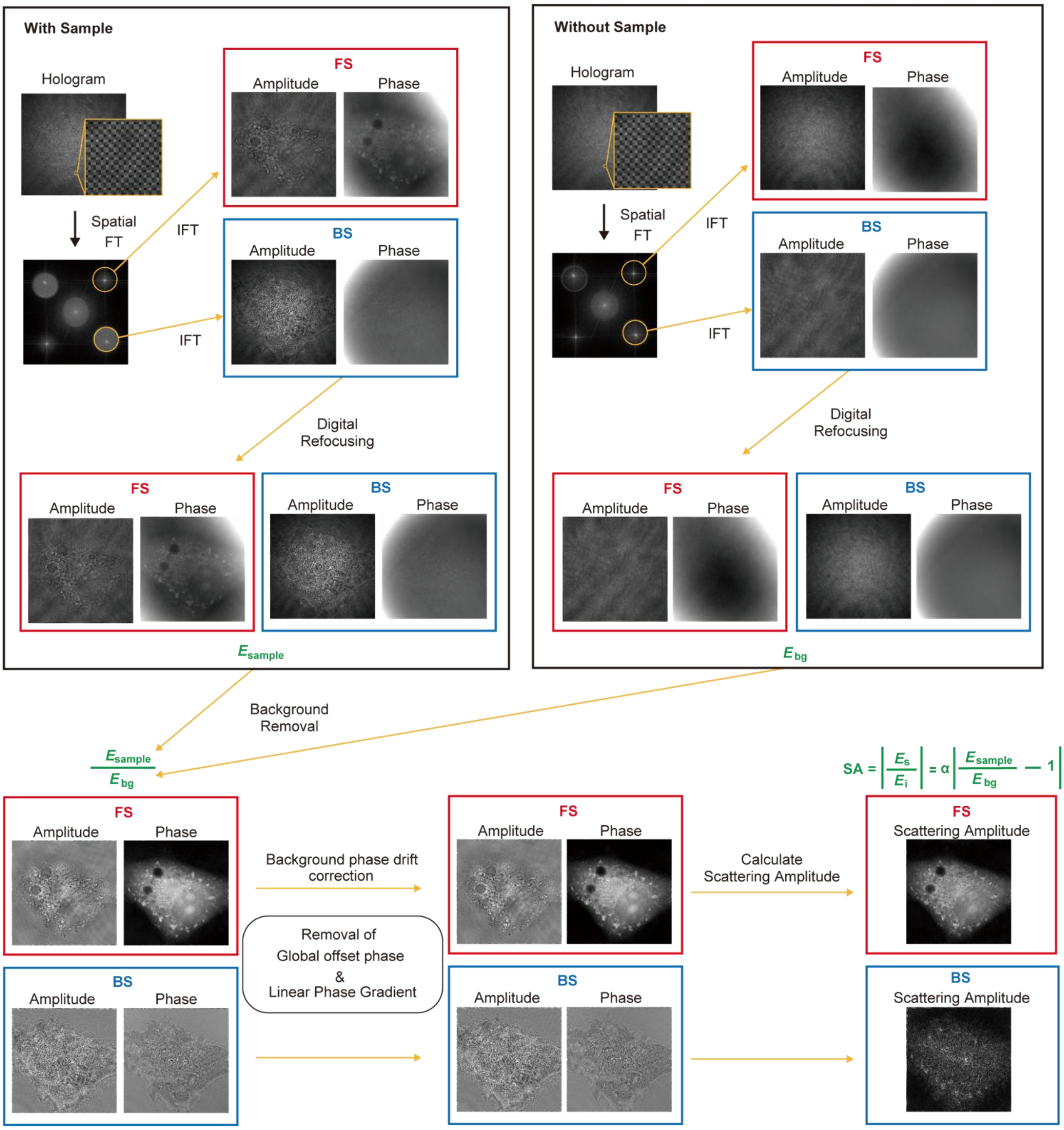

Fig. S3 Computational workflow for generating SA images. FT, Fourier-transform; IFT, inverse Fourier-transform; FS, forward scattering; BS, backward scattering; E_s, complex amplitude of the scattered wave; E_i, complex amplitude of the incident illumination wave; α, a constant defined by $E_{\mathrm{bg}} = \alpha E_{\mathrm{i}}$, representing the field transmittance (FS) or reflectivity (BS) of the glass sample holder.

Supplementary Note 3: FS-BS correlation analysis for determining refractive index and size of small particles. Figure S4a shows the measured FS/BS cross-section ratio plotted against the FS cross-section for silica beads (orange dots), along with theoretical curves for spherical objects with representative sizes and RIs (green dashed and solid reddish curves). In the theoretical calculations, scattering cross-sections were obtained by integrating the Poynting vector derived from the analytical solution in Mie scattering of spherical objects, evaluated along the lateral axes at a fixed axial position. To reduce systematic errors in the measured cross-sections caused by imperfections in the optical system, the cross-sections of the silica beads were normalized by the mean cross-section of polystyrene beads (blue dots). Similarly, the theoretical curves were normalized by the cross-section of a polystyrene bead with a manufacturer-specified RI of 1.59 and a diameter of 151 nm. Figure S4b presents a mapping of RIs and sizes derived from the measured cross-sections of the silica beads. The RI and size were evaluated to be 1.426 ± 0.006 and 206 ± 18 nm, respectively, in close agreement with the manufacturer's specifications of 1.43 and 203 ± 12 nm. The primary contributor to the measured size variation (± 18 nm) is the intrinsic size variation of the beads themselves, specified by the manufacturer as ± 12 nm.

Another contributing factor to the measured size variation is likely the variation in the BS signal caused by surface roughness of the glass coverslip. BS signal variation exhibits a strong dependence on the signal intensity of the beads. The primary source of this variability is spatial heterogeneity in the reflectivity of the coverslip, expressed as $\alpha' = \alpha + \Delta\alpha(x, y)$, which arises from surface roughness—a well-known limitation in iSCAT measurements[1]. This non-uniform reflectivity perturbs the measured SA as:

$$SA' = \frac{\alpha}{\alpha'}\left|\frac{E_s}{E_i}\right| \sim SA(1 - \frac{\Delta\alpha(x,y)}{\alpha}), \quad (1)$$

where SA' denotes the perturbed scattering amplitude, and E_s and E_i are the scattered and incident illumination fields, respectively. The resulting error in the scattering cross-section, $|SA'|^2 - |SA|^2$, scales with the intrinsic BS cross-section magnitude ($|SA|^2$). Indeed, the mean BS cross-section and its variance for polystyrene beads are larger than those for silica beads by similar factors of 3.6 and 5.9, respectively, confirming that BS cross-sectional error is nearly proportional to the signal intensity. Using substrates with reduced surface roughness would be an effective approach to mitigate this error[2]. It should be noted that the above considerations apply exclusively to BS measurements. In contrast, FS signal variations are primarily driven by temporal fluctuations in background FS signals caused by out-of-focus beads. As a result, this variation is independent of the intrinsic FS intensity of the target beads.

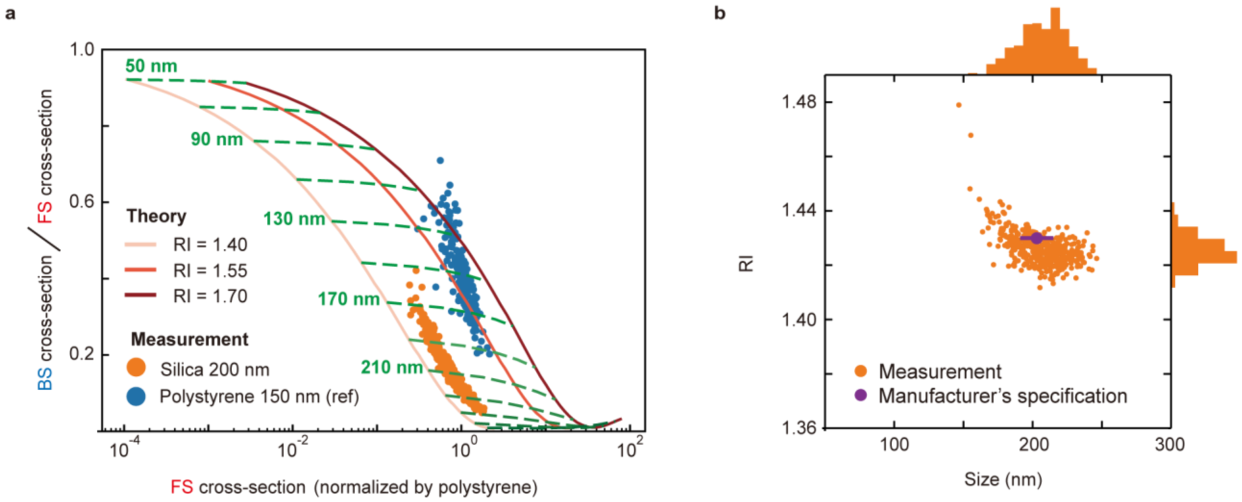

Fig. S4 FS-BS correlation analysis of beads for determining RI and size. **a** FS/BS cross-section ratio plotted against FS cross-section for silica beads. Orange dots represent measured values for silica beads (203 ± 12 nm, 412 particles), whereas the green dashed curves and solid reddish curves show theoretical predictions for spherical objects of varying sizes and RIs in an aqueous environment. The FS cross-section values are normalized by the mean measured cross-section for polystyrene beads (151 ± 3 nm, 204 particles, represented as blue dots). **b** RI and size mapping derived from the measured cross-sections of silica beads. The purple bar represents the manufacturer-specified size variation of the beads (± 12 nm).

Supplementary Note 4: Intracellular particle characterization.

Figure S5a presents the measured FS/BS cross-section ratio against the FS cross-section for the intracellular particles shown in Fig. 3c. These particles exhibit larger FS cross-sections and lower BS/FS ratios compared to those in Fig. S4, indicating larger particle sizes. Figure S5b presents the RI and size estimated for each particle. For particles with diameters below 250 nm, RI and size can be uniquely determined. In contrast, larger particles may yield two or three plausible (RI, size) solutions. For example, Particle 4 has two candidate pairs: (RI, size) = (1.47, 470 nm) and (1.44, 570 nm). Particle 2 presents three possibilities: (RI, size) = (1.41, 503 nm), (1.49, 297 nm), and (1.52, 272 nm). However, the spatial broadening observed in the FS image excludes the first candidate, leaving the latter two, which are plotted in Fig. S5b. All remaining particles exhibit a unique (RI, size) pairing. The extracted values are consistent with those expected for lipid droplets.

In the FS–BS correlation analysis of intracellular particles, the accuracy of FS cross-section measurements is limited by the spatial heterogeneity in the static background, arising from substrate roughness and other intracellular structures. This background introduces an uncertainty of ~0.02 in SA, as FS images are analyzed without temporal differential processing. However, since the FS cross-sections of the Mie-scattering particles examined here are an order of magnitude larger than those in Fig. S4, the relative error from background fluctuations is reduced to ~10%. In contrast, BS cross-sections were obtained through temporal differential analysis and are comparable in magnitude to those of polystyrene beads shown in Fig. S4, resulting in similar variation levels (~15%). Accordingly, we added

error bars to Fig. 3c and S5a by calculating the FS cross-section with a fixed SA uncertainty of 0.02 and BS cross-sections with an SA uncertainty equal to 15% of the measured signal.

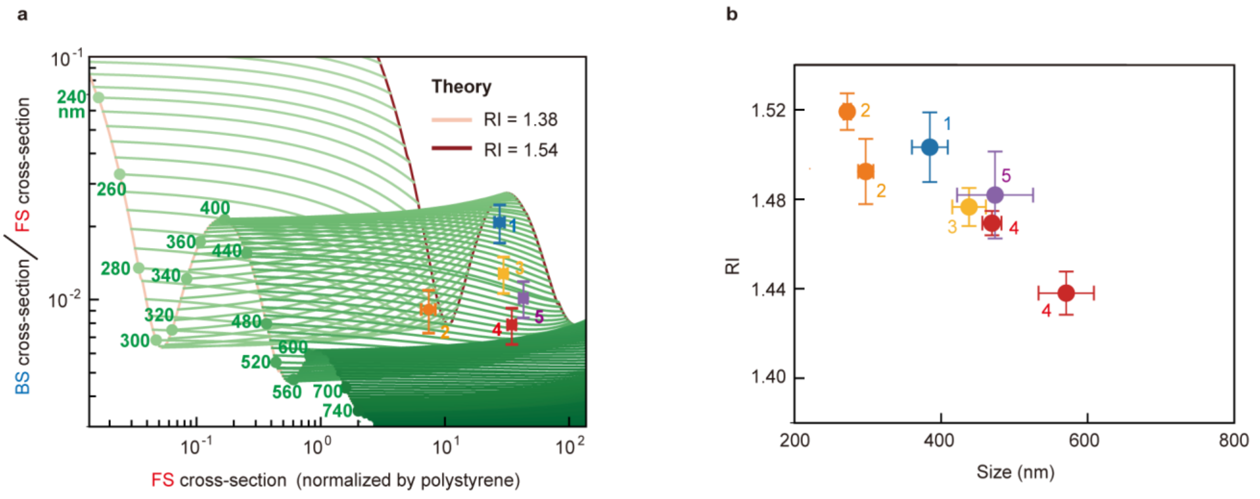

Fig. S5 FS-BS correlation analysis of intracellular particles for determining RI and size. **a** FS/BS cross-section ratio plotted against FS cross-section for Particle 1-5. Dots (blue, orange, yellow, red, and purple) represent measured values, whereas the green curves and reddish curves show theoretical predictions for spherical objects of varying sizes and RIs in the cytoplasm (RI = 1.37). The error bars indicate the measurement accuracy in standard deviation, estimated from the experimental variance in Fig. S4. **b** RI and size mapping derived from the measured cross-sections of intracellular particles. The likelihood function was evaluated, and the corresponding 1σ confidence intervals were depicted as error bars.

Supplementary Note 5: Calculation procedure for dynamic images.

Figure S6a represents the temporal differential image obtained by comparing SA images at t = 0 s and t = 10 s. This differential image reveals a global change in E_{bg} as well as localized differential signals attributed to the movements of intracellular structures. Figure S6b illustrates the procedure used to calculate the dynamic SA image for each imaging pixel.

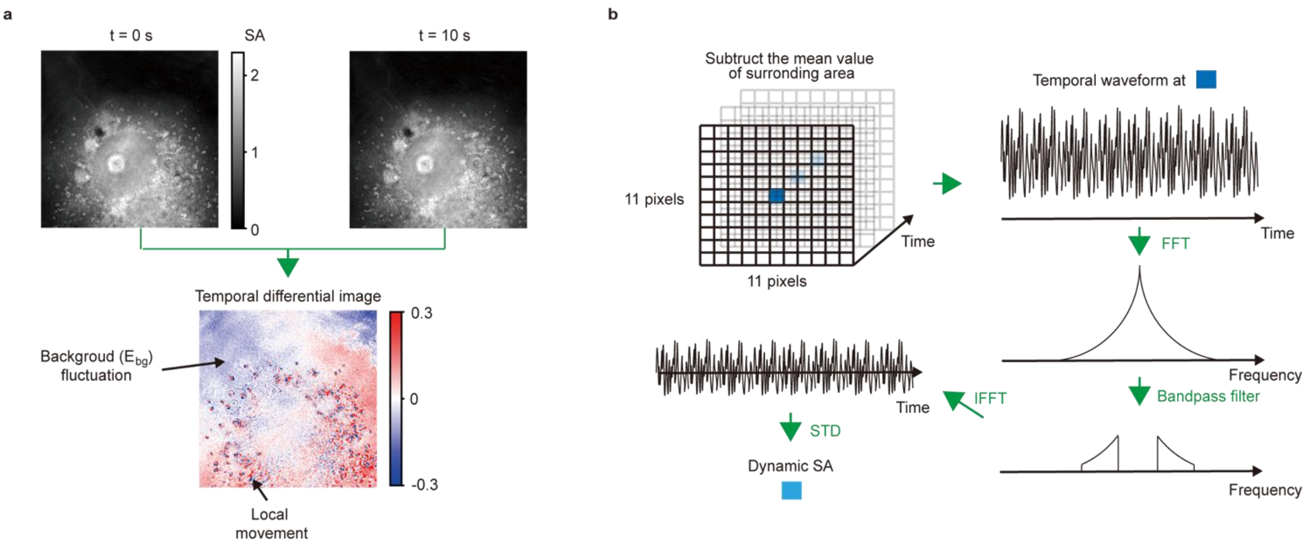

Fig. S6 Long-term temporal variations in background images and procedure for dynamic SA calculation. a SA images at t = 0 s and t = 10 s, and their differential image. **b** Procedure for calculating dynamic SA for each image pixel. FFT: Fast Fourier transform. IFFT: inverse fast Fourier transform. STD: standard deviation.

Supplementary Note 6: Time-lapse observation of cells in the cellular dying process.

Figures S7-S9 present time-lapse imaging data from three additional cells measured under the same conditions as those shown in Fig. 4 of the main manuscript. These results demonstrate consistent temporal trends across samples, supporting the reproducibility of the observed dynamic changes during the cellular dying process.

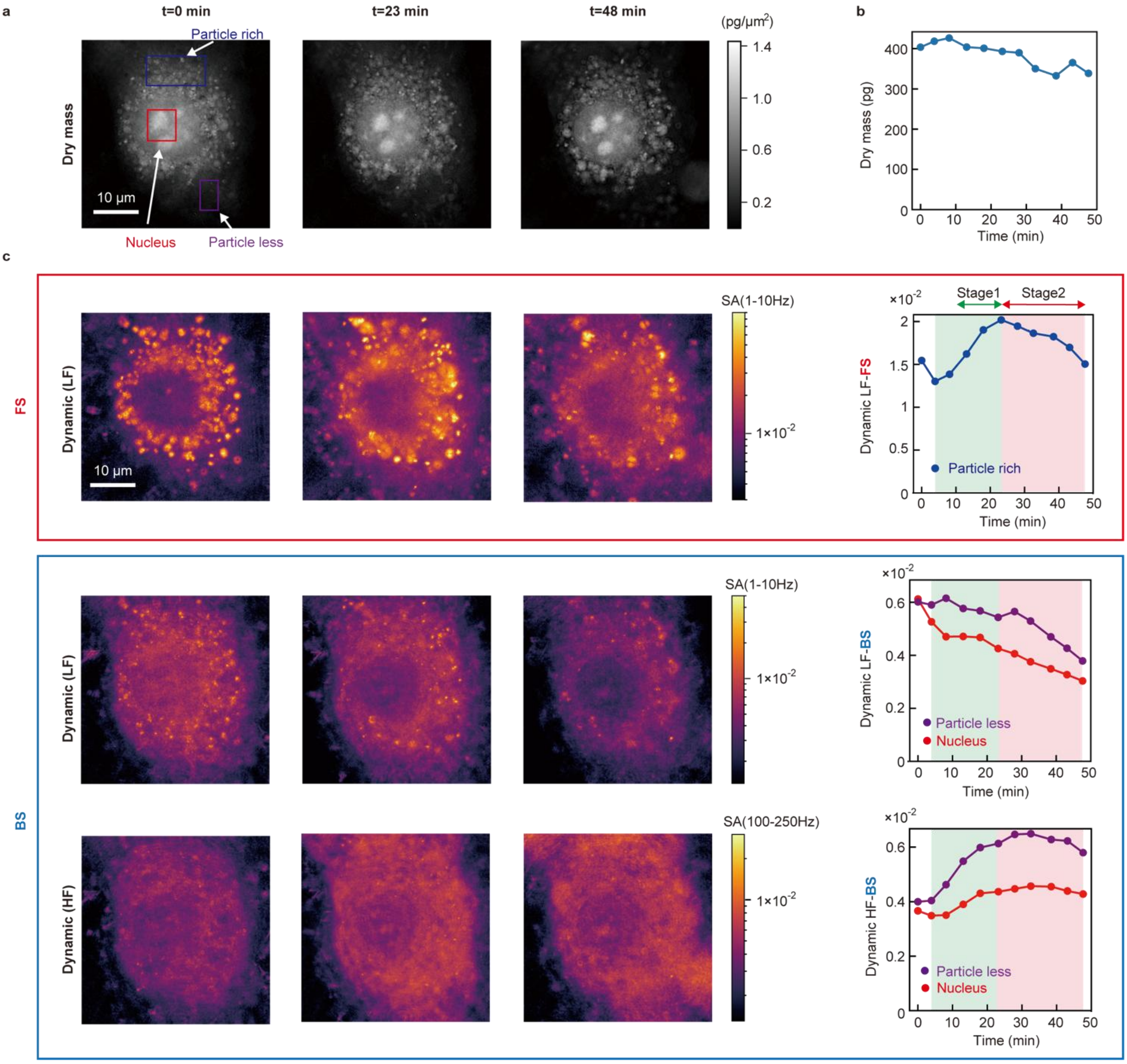

Fig. S7 Time-lapse observation of cell1. a Depth-integrated dry mass concentration maps derived from QPM phase images at 0, 23, and 48 min. **b** Temporal variations of the total dry mass within the cell. **c** Low-frequency (LF)-FS, Dynamic LF-BS, and Dynamic high-frequency (HF)-BS images at 0, 23, and 48 min, respectively (left), and their temporal evolutions of dynamic signals within the representative regions (right): A particle-rich region within the cytoplasm for dynamic FS images (blue), and less-particle cytoplasmic and nuclear regions (purple and red) for dynamic BS images.

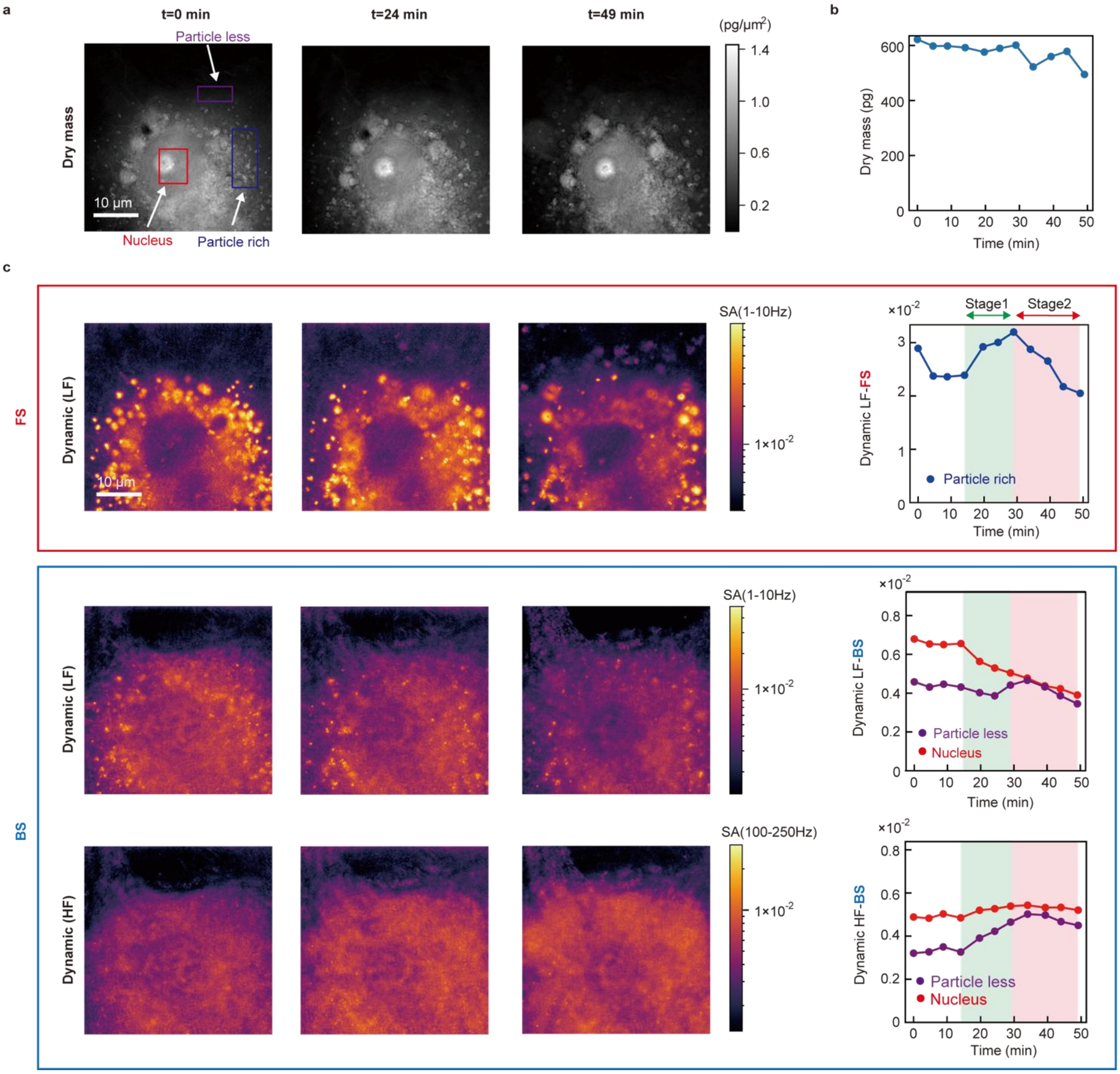

Fig. S8 Time-lapse observation of cell2. a Depth-integrated dry mass concentration maps derived from QPM phase images at 0, 24, and 49 min. **b** Temporal variations of the total dry mass within the cell. **c** Dynamic LF-FS, Dynamic LF-BS, and Dynamic HF-BS images at 0, 24, and 49 min, respectively (left), and their temporal evolutions of dynamic signals within the representative regions (right): A particle-rich region within the cytoplasm for dynamic FS images (blue), and less-particle cytoplasmic and nuclear regions (purple and red) for dynamic BS images.

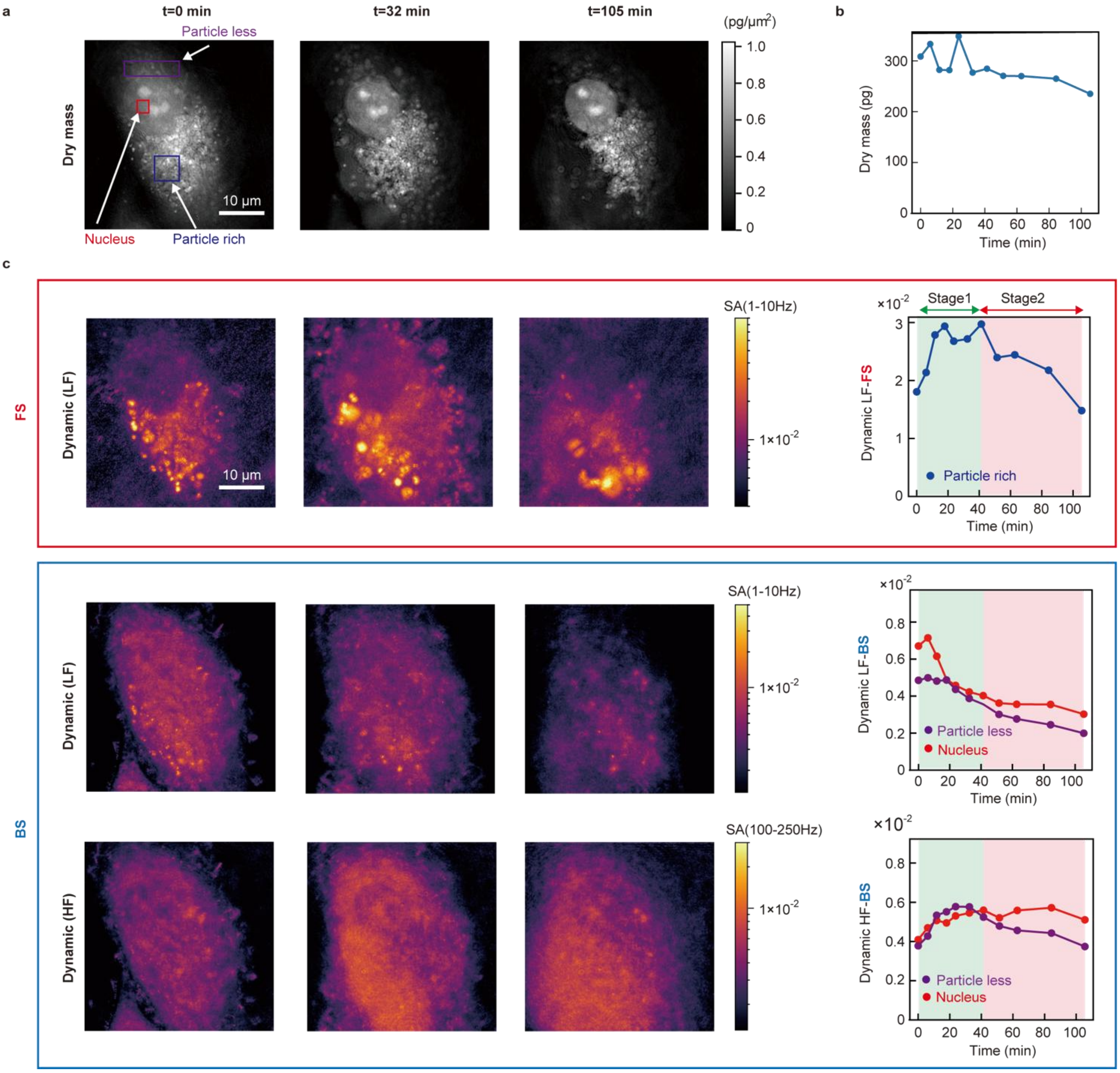

Fig. S9 Time-lapse observation of cell3. a Depth-integrated dry mass concentration maps derived from QPM phase images at 0, 32, and 105 min. **b** Temporal variations of the total dry mass within the cell. **c** Dynamic LF-FS, Dynamic LF-BS, and Dynamic HF-BS images at 0, 32, and 105 min, respectively (left), and their temporal evolutions of dynamic signals within the representative regions (right): A particle-rich region within the cytoplasm for dynamic FS images (blue), and less-particle cytoplasmic and nuclear regions (purple and red) for dynamic BS images.

References.

1. Piliarik, M. *et al.* Direct optical sensing of single unlabelled proteins and super-resolution imaging of their binding sites. *Nat. Commun.* **5,** 4495 (2014).
2. Holanová, K. *et al.* Optical imaging and localization of prospective scattering labels smaller than a single protein. *Opt. Laser Technol.* **109**, 323–327 (2019).
